# Assessing the Effects of eHealth Literacy and the Area Deprivation Index on Barriers to Electronic Patient Portal Use for Orthopedic Surgery: Cross-Sectional Observational Study

**DOI:** 10.2196/72035

**Published:** 2026-01-07

**Authors:** Audrey Lynn Litvak, Nicholas Lin, Kelly Hynes, Jason Strelzow, Jeffrey G Stepan

**Affiliations:** 1 Pritzker School of Medicine, University of Chicago, 924 E 57th Street #104, Chicago, IL, 60637, United States; 2 Department of Orthopaedic Surgery and Rehabilitation Services, University of Chicago, Chicago, IL, United States; 3 Department of Orthopaedic Surgery, Washington University in St Louis, St Louis, MO, United States

**Keywords:** electronic health records, eHealth literacy, online systems, health equity, social determinants of health, SDOH, orthopedic surgery

## Abstract

**Background:**

As electronic patient portals (EPPs) continue to gain popularity and systems transition to online tools for scheduling, communication, and telehealth, patients without access or skills to use these tools may be overlooked.

**Objective:**

This study analyzed patient and neighborhood-level factors, including eHealth literacy level and the Area Deprivation Index (ADI), that may limit EPP access for orthopedic surgery.

**Methods:**

A cross-sectional, survey-based study was performed at a single urban tertiary academic medical center in the United States across foot and ankle, hand and upper extremity, and orthopedic trauma subspecialty clinics from June 21, 2022, to August 12, 2022. Survey responses (N=287) provided information on sociodemographic characteristics; barriers to EPP use and frequency of EPP use; the eHealth Literacy Scale; and the ADI, which is an address-generated national census measure of neighborhood-level disadvantage. Barriers to EPP use were inductively coded into barrier types, classified as physical access, technology discomfort, or preference. The primary outcome measure was patient-reported barriers to EPP use, which was treated as a binary outcome (1=barrier; 0=no barrier). Bivariate analyses and multivariable binary logistic regressions were performed.

**Results:**

The percentage of patients who self-reported barriers to EPP access was 43.2% (124/287), which related to physical access (13/124, 10.4%), technology discomfort (55/124, 44.3%), and preference (78/124, 63.0%). In the adjusted regressions, only low eHealth literacy and older age predicted barriers to EPP use (low eHealth literacy, adjusted odds ratio [AOR] 1.32, 95% CI 1.13-1.54; *P*<.001; older age, AOR 1.007, 95% CI 1.003-1.009; *P*<.001), including barriers of technology discomfort (low eHealth literacy, AOR 1.25, 95% CI 1.11-1.40; *P*<.001; older age, AOR 1.004, 95% CI 1.002-1.007; *P*<.001) and preference (low eHealth literacy, AOR 1.33, 95% CI 1.17-1.51; *P*<.001; older age, AOR 1.004, 95% CI 1.00-1.01; *P*<.01). Patients with physical access–related barriers as opposed to technology discomfort or preference barriers had the lowest median eHealth literacy scores (17.0, IQR 12.0-14.0 vs 27.0, IQR 16.0-32.0 vs 27.0, IQR 20.0-32.0, respectively) and roughly a quartile higher median ADI (73.0, IQR 41.0-92.0 vs 53.5, IQR 31.2-76.0 vs 58.0, IQR 38.8-83.8, respectively).

**Conclusions:**

Low eHealth literacy was the most significant determinant of overall barriers to EPP use for orthopedic surgery, followed by older age. Neighborhood-level disadvantage as measured through the ADI had no mediating effect on patient-reported barriers to EPP use when adjusting for eHealth literacy level. While patients with physical access barriers had higher ADIs, overall, few patients reported physical access barriers compared to barriers related to technology discomfort or preference. Patient preference for EPP versus non-EPP communications should be documented. Point-of-care screening using the eHealth Literacy Scale may also identify patients who require follow-up outside of the EPP during critical perioperative periods.

## Introduction

Improving digital health information transparency and transmission via electronic patient portals (EPPs) has been a central focus of health IT policy in the United States during the last decade [1]. EPPs facilitate patients’ communication with their treating care teams and direct access to their electronic personal health record and tools to request prescription refills, participate in e-visits, and complete patient-reported outcome questionnaires, among other functions. These interactions empower patients to take an active role in their health care [1,2]. However, few studies have examined patient portal use in the surgical setting or patient factors that may limit EPP use in this context [3-8].

Benefits of EPP enrollment among orthopedic patients include improved patient outcomes [4,7], medication adherence [3], higher patient satisfaction and psychosocial health [3,7], and fewer missed appointments [3]. Patient engagement via the EPP may additionally facilitate more effective screening for commonly avoidable complications that delay patients’ return to function, such as soaking of splints or patient-prolonged immobilization due to unanticipated postoperative pain. Moreover, as routine messaging and completion of patient-reported outcomes via the EPP becomes standard, it is likely that patient engagement via the EPP beyond enrollment may become another critical quality metric tied to physician reimbursement.

Despite advantages of and health care provider interest in adoption of EPP tools, prior studies in orthopedic surgery have shown that patient factors, including older age and lower educational level, may limit EPP enrollment [3,4,6,8], which is analogous to observations in the internal medicine setting [9-12]. The 2020 Health Information National Trends Survey (HINTS) found that the most commonly cited reason for patient nonuse within a large US sample was desire to speak directly with a health care provider (ie, physician, nurse practitioner)—a sentiment shared by 69% of patients [11]. Furthermore, roughly 30% of patients expressed discomfort with the technology [11]. Traditional health literacy refers to the capacity to find, understand, and use health information to inform health-related decisions and actions, whereas eHealth literacy specifically refers to the capacity and skills to seek, assess, and make use of health information via electronic media. In the hospital medicine setting, low eHealth literacy in particular is associated with less awareness, use, and perceived usefulness of EPPs [13].

Lack of examination of granular patient-level use data beyond EPP activation status, explicit barriers to EPP use, and associated patient factors such as health literacy are described as significant limitations and directions for future work in orthopedic surgery [3,4,8]. Additionally, no study across any prior setting has assessed the effect of structural or neighborhood-level determinants on barriers to EPP use, nor have they assessed barriers among patients who are actively enrolled in EPPs. Individual-level determinants may refer to patient demographics or skill sets such as health literacy, whereas neighborhood-level determinants refer to unmeasured social factors conferred by the geographic environment in which a patient lives, often described via census variables related to percentage of unemployment, percentage of individuals with a high school education, and food and housing quality, among others.

For digital health uptake in particular, distinguishing among types and levels of determinants is critical to realizing equity-informed intervention and policy [14,15]. For example, digital literacy is an individual-level factor for which a policy-level solution such as improving broadband connectivity or personal device accessibility may be ineffective in the absence of community-responsive interventions to provide individuals with digital skill training [15]. In particular, it is important to analyze whether neighborhood disadvantage may amplify the impact of low eHealth literacy on barriers to patient portal use, which single-level analyses of eHealth literacy cannot capture.

This study aimed to contribute to the existing body of literature on barriers to EPP use in orthopedic surgery by analyzing how both individual-level and neighborhood-level social determinants, including eHealth literacy and the Area Deprivation Index (ADI), may relate to patient-reported barriers to EPP access and use across foot and ankle, hand and upper extremity, and orthopedic trauma surgery.

## Methods

### Study Design and Setting

This was a cross-sectional survey–based study conducted via an anonymized paper survey administered at a single urban tertiary academic medical center in the United States between June 21, 2022, and August 12, 2022. The survey was administered in the clinic following each patient visit and consisted of sociodemographic questions, the eHealth Literacy Scale (eHEALS), and 2 questions regarding EPP access and use detailed below. This study followed the Strengthening the Reporting of Observational Studies in Epidemiology (STROBE) design and reporting guidelines for cross-sectional studies.

### Participants

All English-speaking patients aged >18 years presenting for orthopedic surgery evaluation at foot and ankle, trauma, and hand and upper extremity clinics were included and approached consecutively during the aforementioned period. This study was limited to foot and ankle, trauma, and hand and upper extremity surgery clinics where faculty involvement at our institution was feasible. Approached patients were excluded if they had not received a tablet to complete their in-office patient-reported outcome questionnaire per routine standard of care due to external technological or capacity constraints (ie, tablet out of battery or too few working tablets in the clinic on a particular day) unless that patient had already completed the questionnaire via their patient portal. While this practical limitation resulted in potential selection bias, it randomly affected only a small portion of patients (<10) and was remedied to avoid recurrence of the problem for continued recruitment efforts. Patients who could not read or write were included and read aloud the study survey.

### Sample Size Calculation

The HINTS 2020 reported that approximately 40% of adults in the United States accessed their patient portal in the previous year, whereas 59% were nonusers [11]. Using a baseline nonuse or potential barrier rate of roughly 40% to 60% and setting an α value of .05, we estimated that a sample of 270 to 290 patients would provide at least 80% power to detect large effect sizes (odds ratio 2.0‐3.0) in a binary regression model constrained by 10 events per covariate included.

### Ethical Considerations

Biological Sciences Division/University of Chicago Institutional Review Board approval (IRB22-0230) was obtained with waivers for written consent and HIPAA (Health Insurance Portability and Accountability Act) authorization. Anonymized survey data were transcribed into a REDCap (Research Electronic Data Capture; Vanderbilt University) database for secure storage [16]. No compensation was provided for participation.

### Primary and Secondary Outcomes

The primary outcome measure was patient-reported barriers to EPP access. Barriers to access were derived from the HINTS and secondarily classified as barriers of physical access, discomfort with technology, or patient preferences for nonelectronic provider communication (Multimedia Appendix 1) [11]. These categories were inductively coded by the research team after data collection. This classification is not validated, which we discuss as a limitation. Patients were instructed to mark preference for nonelectronic provider communication as a selection only if they perceived their preferences as barriers to using their portals. Importantly, “I do not have a patient portal account” was listed as an option on the original survey but was analyzed separately. The secondary outcome measure was patient-reported level of EPP use classified into 2 categories: routine use and nonroutine use. Per Maroney et al [17], level of use was characterized as routine if at least monthly use was indicated and as nonroutine if use a few times a year or less frequently was indicated, including those who did not have an EPP (Multimedia Appendix 1). Importantly, the level of EPP use included use for any clinic, not limited only to their orthopedic surgery care.

### Variables and Demographics

eHealth literacy was measured via patient responses to the eHEALS tool to determine its association with barriers to EPP use (Multimedia Appendix 2) [18]. This tool has been validated in the orthopedic outpatient setting, among others [18-20]. As in prior literature, a cumulative score of 25 or less indicated low eHealth literacy, and a score of 26 or greater indicated high eHealth literacy [13,21,22]. Neighborhood-level disadvantage was assessed using the ADI, which is calculated via publicly available census data in the domains of income, educational level, employment, and housing quality to assign numeric scores of societal disadvantage to particular geographical regions [23]. Higher scores indicate higher levels of societal disadvantage. Self-reported demographic data were additionally collected.

### Statistical Analysis

Survey data were analyzed using the Python statistical program (version 3.10.6; Python Software Foundation) [24-26]. Missing values were excluded pointwise across the relevant analyses given the low frequency. Numerical data presented as medians were reported with the IQR. The level of significance was set at *P*=.05.

Bivariate analyses were performed to examine the association between both patient-reported barriers and level of EPP use and demographic variables, the ADI, and eHealth literacy level. Three multivariable logistic regressions were conducted wherein barriers were treated as a binary outcome (1=barrier; 0=no barrier). Categorical variables were converted to binary dummy variables for regression. The main regression considered all barriers, whereas the 2 subsequent regressions examined only barriers of technology discomfort or preference-related barriers. Regression was not performed for physical access–related barriers due to outcome size of 13, which is discussed as a limitation. Variable selection for each model was determined via outcome size (at least 10 outcomes per covariate to avoid overfitting) and one-at-a-time sensitivity analyses (via examination of the McFadden pseudo-*R*^2^) in a backward regression approach. To avoid collinearity, only covariates with a variance inflation factor of <5 were included together. Model fit was assessed using McFadden pseudo-*R*^2^ values, with 0.2 considered excellent if not overfit (corroborated via df).

Demographic categories and regression reference levels were selected based on breakdowns and historical controls used in prior related literature [9,27,28]. Income was treated categorically, and per the analogous literature, we selected 3 levels representative of low, medium, and high income based on the median household income cutoffs for the zip code tied to the authors’ institution.

## Results

### Participant Characteristics

A convenience sample of 339 eligible patients was approached, of whom 52 (15.3%) declined participation, leaving 287 (84.7%) for analysis. The median age of the study participants was 48.5 (IQR 35.0-64.2) years; 58.2% (167/287) of the study participants self-identified as non-Hispanic Black individuals, and 26.1% (75/287) identified as non-Hispanic White individuals. The median cumulative eHEALS score was 32 (IQR 27-35), with 21.3% (61/287) of the study participants having low eHealth literacy (eHEALS score of 25 or less). The median ADI was 53.0 (IQR 32.0-74.8).

Of the study participants, 63.1% (181/287) were routine users, and only 9.8% (28/287) did not have EPPs. One or more barriers to accessing their EPPs were reported by 43.2% (124/287) of all patients. Moreover, among the 90.2% (259/287) of patients who were enrolled in the EPP, 42.5% (110/259) still reported barriers to access or use of their portal. The remaining patient characteristics were compared by self-reported barriers to EPP access and self-reported EPP use (Table 1). Patients reporting one or more barriers had higher median age than patients who did not report barriers (57.0, IQR 42.2-71.0 years vs 43.0, IQR 29.2-57.0 years; *P*<.001; Table 1). A higher percentage of non-Hispanic Black patients (*P*=.04), retirees (*P*=.001), and patients who fell into the income bracket of US $30,000 or less (*P*=.03) reported barriers to access (Table 1). Patients who did not routinely use the EPP had a lower educational level than routine users (*P*=.005; Table 1).

**Table 1.** Descriptive characteristics and exploratory comparison of self-reported barriers to electronic patient portal (EPP) access and self-reported EPP use by patient characteristics.^a^

Characteristic	Overall	No barriers	One or more barriers	*P* value	Routine use	Nonroutine use	*P* value
Subspecialty clinic, n/N (%)				.07			.77
Hand	187/287 (65.2)	111/187 (59.4)	76/187 (40.6)		116/187 (62.0)	71/187 (38.0)	
Foot and ankle	68/287 (23.7)	40/68 (58.8)	28/68 (41.2)		43/68 (63.2)	25/68 (36.8)	
Trauma	32/287 (11.1)	12/32 (37.5)	20/32 (62.5)		22/32 (68.8)	10/32 (31.3)	
Age (years), median (IQR)	48.5 (35.0-64.2)	43.0 (29.2-57.0)	57.0 (42.2-71.0)	<.001	47.0 (35.0-62.0)	52.0 (37.0-67.0)	.28
Race or ethnicity, n/N (%)				.04			.09
Hispanic or Latino	24/287 (8.4)	16/24 (66.7)	8/24 (33.3)		12/24 (50.0)	12/24 (50.0)	
Non-Hispanic Black	167/287 (58.2)	83/167 (49.7)	84/167 (50.3)		103/167 (61.7)	64/167 (38.3)	
Non-Hispanic White	75/287 (26.1)	51/75 (68.0)	24/75 (32.0)		48/75 (64.0)	27/75 (36.0)	
Other identity or preferred not to answer	21/287 (7.3)	13/21 (61.9)	8/21 (38.0)		18/21 (85.7)	3/21 (14.3)	
Highest educational level attained, n/N (%)				.07			.005
High school or lower	82/284 (28.9)	39/82 (47.6)	43/82 (52.4)		41/82 (50.0)	41/82 (50.0)	
Some college	73/284 (25.7)	40/73 (54.8)	33/73 (45.2)		45/73 (61.6)	28/73 (38.4)	
College or higher	129/284 (45.4)	82/129 (63.6)	47/129 (36.4)		93/129 (72.1)	36/129 (27.9)	
Annual income bracket (US $), n/N (%)				.03			.09
≤30,000	89/260 (34.2)	42/89 (47.2)	47/89 (52.8)		52/89 (58.4)	37/89 (41.6)	
30,001-50,000	48/260 (18.5)	26/48 (54.2)	22/48 (45.8)		30/48 (62.5)	18/48 (37.5)	
>50,000	123/260 (47.3)	80/123 (65.0)	43/123 (35.0)		89/123 (72.4)	34/123 (27.6)	
Current employment status, n/N (%)				.001			.19
Employed	160/285 (56.1)	105/160 (65.6)	55/160 (34.4)		109/160 (68.1)	51/160 (31.9)	
Unemployed or on disability	66/285 (23.2)	35/66 (53.0)	31/66 (47.0)		38/66 (57.6)	28/66 (42.4)	
Retired	59/285 (20.7)	22/59 (37.3)	37/59 (62.7)		34/59 (57.6)	25/59 (42.4)	

a Percentages may not add up to 100 due to rounding.

### Exploratory Analysis of eHealth and ADI as Potential Barriers to EPP Access and Use

In the analysis of eHealth literacy, patients who reported barriers had a lower median eHEALS score than patients who did not report barriers (29.0, IQR 22.0-32.2 vs 32.0, IQR 30.0-38.0; *P*<.001; Table 2). Conversely, a higher percentage of patients with low eHealth literacy compared to high eHealth literacy reported barriers to using their EPPs (45/61, 74% vs 79/224, 35%, respectively; *P*<.001; Table 2). Patients who reported barriers also had higher median national ADI than patients who did not report barriers (55.5, IQR 37.5-78.2 vs 51.0, IQR 32.0-70.0; *P*=.06), and a higher percentage of patients from the most deprived ADI quartile also reported barriers compared to patients from the least deprived ADI quartiles (most deprived ADI quartile [76-100], 37/70, 53% vs second most deprived ADI quartile [51-75], 35/87, 40% vs second least deprived ADI quartile [26-50], 29/70, 41% vs least deprived ADI quartile [1-25], 19/55, 35%; *P*=.19); however, these results did not reach statistical significance (Table 2).

**Table 2.** Comparison of self-reported barriers to electronic patient portal access by eHealth literacy level and the Area Deprivation Index (ADI).^a^

	Overall	No barriers	Any barriers	*P values*
eHEALS^b^ score, median (IQR)	32.0 (27.0-35.0)	32.0 (30.0-38.0)	29.0 (22.0-32.2)	<.001
eHealth literacy level (eHEALS score), n/N (%)				<.001
High eHealth literacy	224/285 (78.6)	145/224 (64.7)	79/224 (35.3)	
Low eHealth literacy	61/285 (21.4)	16/61 (26.2)	45/61 (73.8)	
National ADI, median (IQR)	53.0 (32.0-74.8)	51.0 (32.0-70.0)	55.5 (37.5-78.2)	.06
National ADI quartile, n/N (%)				.19
Least deprived ADI quartile (1-25)	55/282 (19.5)	36/55 (65.4)	19/55 (34.5)	
Second least deprived ADI quartile (26-50)	70/282 (24.8)	41/70 (58.6)	29/70 (41.4)	
Second most deprived ADI quartile (51-75)	87/282 (30.9)	52/87 (59.8)	35/87 (40.2)	
Most deprived ADI quartile (76-100)	70/282 (24.8)	33/70 (47.1)	37/70 (52.9)	

a Percentages may not add up to 100 due to rounding and missing values.

b eHEALS: eHealth Literacy Scale.

Most of the patient-reported barriers were related to technology discomfort (55/124, 44.4%) or preference (78/124, 62.9%) rather than physical access (13/124, 10.5%). However, the group reporting physical access barriers had the lowest levels of eHealth literacy (median eHEALS score 17.0 vs 27.0 vs 27.0, respectively) and highest ADI (median 73.0 vs 53.5 vs 58.0, respectively) compared to groups reporting barriers related to technology discomfort or preference (Table 3). Patients from the most deprived ADI quartile had higher percentages of barriers (Table 3). Bivariate analysis was not performed as barrier type was nonexclusive.

**Table 3.** Exploratory analysis of barrier type by eHealth literacy level and the Area Deprivation Index (ADI).^a^

	No barriers	Physical access barriers	Technology discomfort barriers	Preference barriers
eHEALS^b^ score, median (IQR)	32.0 (30.0-38.0)	17.0 (12.0-24.0)	27.0 (16.0-32.0)	27.0 (20.0-32.0)
eHealth literacy level (eHEALS), n/N (%)				
High eHealth literacy	145/224 (64.7)	2/224 (0.9)	30/224 (13.4)	44/224 (19.6)
Low eHealth literacy	16/61 (26.2)	11/61 (18.0)	25/61 (41.0)	34/61 (55.7)
National ADI, median (IQR)	51.0 (32.0-70.0)	73.0 (41.0-92.0)	53.5 (31.2-76.0)	58.0 (38.8-83.8)
National ADI quartile, n/N (%)				
Least deprived ADI quartile (1-25)	36/55 (65.5)	0/55 (0.0)	10/55 (18.2)	10/55 (18.2)
Second least deprived ADI quartile (26-50)	41/70 (58.6)	4/70 (5.7)	13/70 (18.6)	19/70 (27.1)
Second most deprived ADI quartile (51-75)	52/87 (59.8)	3/87 (3.4)	17/87 (19.5)	15/87 (17.2)
Most deprived ADI quartile (76-100)	33/70 (47.1)	6/70 (8.6)	14/70 (20.0)	30/70 (42.9)

a Percentages may not add up to 100 due to rounding and missing values.

b eHEALS: eHealth Literacy Scale.

### Regression Analysis of Barriers to EPP Use

In the overall regression including demographic variables, the ADI, and eHealth literacy level, only low eHealth literacy level (adjusted odds ratio [AOR] 1.32, 95% CI 1.13-1.54; *P*<.001) and older age (AOR 1.007, 95% CI 1.003-1.009; *P*<.001) predicted barriers to EPP access (Table 4). Similarly, only low eHealth literacy level and age were associated with predicting a technology discomfort–related barrier (low eHealth literacy, AOR 1.25, 95% CI 1.11-1.40; *P*<.001; age, AOR 1.004, 95% CI 1.002-1.007; *P*<.001; Table 4) or a preference-related barrier (low eHealth literacy, AOR 1.33, 95% CI 1.17-1.51; *P*<.001; age, AOR 1.004, 95% CI 1.00-1.01; *P*=.01; Table 4). The ADI was not associated with predicting overall barriers (*P*=.59), preference-related barriers (*P*=.35), or technology discomfort–related barriers (*P*=.76; Table 4).

**Table 4.** Regression of patient characteristics associated with self-reporting at least one barrier to electronic patient portal use (any barrier type, preference barrier, or technology discomfort barrier).

Characteristic	Regression 1: any barrier type^a^		Regression 2: preference barriers^b^		Regression 3: technology discomfort barriers^c^	
	AOR^d^ (95% CI)	*P* value	AOR (95% CI)	*P* value	AOR (95% CI)	*P* value
Age	1.007 (1.003-1.009)	<.001	1.004 (1.00-1.01)	.01	1.004 (1.002-1.007)	<.001
Race or ethnicity						
Hispanic or Latino	0.99 (0.78-1.25)	.92	1.07 (0.88-1.31)	.49	1.04 (0.86-1.24)	.71
Non-Hispanic Black	1.09 (0.94-1.27)	.26	1.09 (0.95-1.24)	.21	1.04 (0.92-1.17)	.52
Non-Hispanic White	Reference	—^e^	Reference	—	Reference	—
Other identity or preferred not to answer	1.02 (0.79-1.32)	.88	1.05 (0.85-1.30)	.63	0.95 (0.78-1.15)	.56
National ADI^f^	1.00 (1.00-1.00)	.59	1.00 (1.00-1.00)	.35	1.00 (1.00-1.00)	.76
eHealth literacy level (eHEALS^g^)						
High eHealth literacy	Reference	—	Reference	—	Reference	—
Low eHealth literacy	1.32 (1.13-1.54)	<.001	1.33 (1.17-1.51)	<.001	1.25 (1.11-1.40)	<.001
Subspecialty clinic						
Hand	Reference	—	Reference	—	—	—
Foot and ankle	1.01 (0.88-1.16)	.91	1.02 (0.91-1.15)	.73	—	—
Trauma	1.18 (0.99-1.41)	.07	1.17 (1.00-1.37)	.05	—	—
Highest educational level attained						
High school or lower	1.06 (0.90-1.25)	.65	—	—	—	—
Some college	0.96 (0.82-1.13)	.51	—	—	—	—
College or higher	Reference	—	—	—	—	—
Annual income bracket (US $)						
≤30,000	1.11 (0.94-1.30)	.22	—	—	—	—
30,001-50,000	1.05 (0.89-1.24)	.59	—	—	—	—
>50,000	Reference	—	—	—	—	—

a Regression model 1: 88.5% (254/287) of the patients were included after participants with missing values were excluded (outcome size: 124/254, 48.8% reported any barrier; df=12; pseudo*-R*2=0.21).

b Regression model 2: 96.5% (277/287) of the patients were included after participants with missing values were excluded (outcome size: 78/277, 28.2% reported preference barriers; df=8; pseudo*-R*2=0.16).

c Regression model 3: 96.5% (277/287) of the patients were included after participants with missing values were excluded (outcome size: 55/277, 19.9% reported technology discomfort barriers; df=6; pseudo*-R*2=0.13).

d AOR: adjusted odds ratio.

e Not applicable.

f ADI: Area Deprivation Index.

g eHEALS: eHealth Literacy Scale.

**Figure 1.**
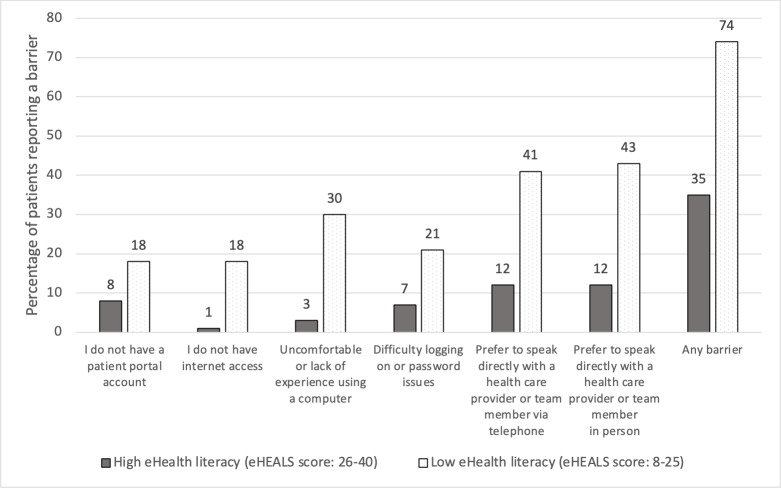
Frequency and percentages of patient self-reported barriers to electronic patient portal (EPP) access stratified by eHealth literacy level. The percentages of patients who reported a particular barrier are reported over the denominator of patients with either high eHealth literacy scores (n=224) or low eHealth literacy scores (n=61). Percentages do not necessarily add up to 100 across barrier categories as patients could indicate more than one barrier. The barriers “I do not need a patient portal account,” “I have multiple patient portals,” and “I have privacy concerns” are not depicted separately due to lower frequency of observations.

The most prevalent patient-reported barrier to EPP access was a preference to speak directly to the health care provider or team member in person or via telephone (52/287, 18.1%). A lack of comfort with a computer was cited as a barrier to EPP access by 29.5% (18/61) of patients with low eHealth literacy (Figure 1). Additionally, 17.8% (51/287) of study participants reported poor or fair ability to use a computer, tablet, or smartphone to find information that they needed on the internet. One or more barriers to EPP use were indicated by 80.4% (41/51) of patients with poor or fair self-ratings, compared to 35.3% (83/235) of patients with good, very good, or excellent self-ratings (*P*<.001).

## Discussion

### Principal Findings

Equitable implementation of digitized health tools relies on efforts from clinicians, researchers, and policymakers alike. This study assessed patient-reported barriers to use within an expanded framework of individual- and neighborhood-level factors. Low eHealth literacy level was the most significant determinant of overall barriers to EPP use for orthopedic surgery, followed by older age, as compared to other demographic factors and measures of neighborhood-level disadvantage. Contrary to expectation, neighborhood-level disadvantage as measured via the ADI had no mediating effect on barriers after adjusting for eHealth literacy level. Patients with physical access barriers did have appreciably higher ADIs; however, few patients reported physical access barriers overall. These findings build on prior work that showed that older age, among other patient demographics, was associated with reduced EPP enrollment in orthopedic surgery [3,4,8]. This also builds on prior work in the hospital medicine setting that showed that lower eHealth literacy correlated with decreased awareness and use and less favorable attitudes toward use of EPPs [13].

Barriers of physical access, such as lack of internet access, were infrequent compared to barriers related to preference or discomfort with technology, including lack of experience using a computer or difficulty logging in. This finding contrasts with previous work that found internet access to be a significant determinant of portal use for orthopedic surgery [6]. Additionally, among the 17.8% (51/287) of the participants with lower self-ratings of ability to use a computer, tablet, or smartphone to find information that they needed on the internet, 80.4% (41/51) indicated barriers to accessing their EPPs. The final rule of the 21st Century Cures Act alleviated many barriers related to physical access (eg, computer access or broadband coverage) by requiring an interoperability standard that any EPP programming interface be compatible with smartphone apps [29]. However, this legislation does not address barriers experienced by patients who may technically have the physical and digital tools to access their EPPs but not the self-efficacy or the skill sets to effectively use these tools.

These findings may support a second digital divide being dependent on disparities in skill sets rather than physical access [13,30]. With regard to EPPs, this is clinically significant as most institutions introduce EPPs via email with time-sensitive links and sign-up instructions. This may be ineffective at best in promoting EPP adoption or postenrollment use in populations who may have internet access yet lack the internet experience and skills to navigate setting up a digital account with time-pressure activation codes for protected health information. Moreover, the finding that over a quarter of all patients (52/287, 18.1%) perceived their preference to speak with a health care provider in person or via telephone as an actual barrier to using the EPP suggests that patients may view the EPP as a substitute for health care provider communication missing the personal element rather than as an adjunct to improve communication and transparency, as it was intended. This finding also suggests that it may be important to document patient preference for EPP versus non-EPP communication even if a patient does have an EPP as patients with activated EPPs may not be active users.

Importantly, while most patients were enrolled in the EPP, a significant portion of enrolled patients reported barriers to access and use of their portal. This is significant as prior studies of EPP use for orthopedic surgery have either only assessed EPP activation status as a surrogate for use and without assessing barriers to use [3,4,8] or qualitatively analyzed optional comments regarding nonuse in a small fraction of the study cohort (38 of 150 patients) [6]. These findings are clinically relevant as prior efforts to reduce disparities in EPP use have also focused on enrollment [9]. However, simply enrolling patients does little to address the underlying barriers of certain patients to using their EPPs. Enrollment will not address individual-level factors such as eHealth literacy, which may constitute a larger underlying barrier to sustained EPP use after enrollment (eg, patients’ technological capabilities and skills rather than physical access to the technology itself).

Additionally, this study substantiates that older adults are a vulnerable population that may be left behind in a digitized health system. This is particularly critical to perioperative care in orthopedic surgery. Older patients may have more complex discharge needs, including perioperative medication changes and rehabilitation requiring close postoperative communications, and addressing them within the EPP may be ineffective. Older patients have intersecting factors that impede their access. While age and eHealth literacy were noncollinear in this study, age has well-known associations with traditional health and eHealth literacy [31]. Addressing deficiencies in these skill sets may improve lower levels of self-efficacy to adopt and use EPPs previously reported in older individuals [32]. This group may benefit from a proactive staff-level intervention that supports an in-person EPP enrollment option followed by an initial lesson on how to use the EPP, which Bhashyam et al [33] previously showed may be beneficial to improving postoperative follow-up. Notably, while older patients may have an elevated sense of caution in using online platforms due to counseling from groups such as the American Association of Retired Persons, patients infrequently noted privacy concerns as a barrier to EPP use in this study.

Patients with physical access barriers also had appreciably higher ADIs despite this analysis not meeting statistical significance. These patients may also benefit from routine touchpoints with staff outside of the EPP. Ensuring effective follow-up is especially important in these patients as, in addition to worse ADIs predicting worse comorbid chronic disease outcomes, simply living in a disadvantaged neighborhood confers similar readmission risk as having a chronic lung disease and higher risk than having a chronic condition such as diabetes [34]. At an informatics design level, a widget within the electronic medical record could be implemented to automatically yield an address-generated ADI analogously to how BMI is automatically calculated based on a patient’s weight and height as this may generate similarly important contextual information to a patient’s overall health, especially in a perioperative context.

### Limitations

First, this was a single-institution study conducted across several orthopedic surgery subspecialty clinics not including adult reconstruction. Hence, the results may not be generalizable across other settings. Moreover, while eHEALS is a commonly used, validated screener for eHealth literacy in outpatient settings [18-20], it has not been validated in our specific patient population. Additionally, self-reporting via a survey may be limited by response bias; however, we felt that this method of examination was critical to include the patient perspective. Importantly, this study did not include non–English-speaking patients, who may experience additional barriers to EPP access; however, this study did include the perspectives of patients with limited reading and writing skills.

Additionally, level of use was dichotomized as routine and nonroutine, similar to the study by Maroney et al [17], without an option for “as needed,” which assumes regular use. The National Cancer Institute’s 2020 HINTS showed that only 40% of those with EPPs used it every year: 65% felt that they did not need to use it every year [11]. An additional checkbox option for “use as needed” may better capture this nuance; however, this could introduce indeterminate subjectivity.

Notably, the sample size may have been underpowered to detect small effect sizes in demographic differences and in novel outcomes such as the ADI. The sample size calculation was predicated on the 2020 HINTS, which analyzed barriers to EPP enrollment and did not include patients with activated EPPs who still experienced barriers (259/287, 90.2% of our study population) [11]. Moreover, no relevant existing literature has assessed the ADI. Finally, the secondary barrier categories were inductively coded by our research team after data collection based on natural groupings in which we were interested. This rendered our initial sample size calculation insufficient to perform a secondary regression analysis for the access-related barrier category, which had a small outcome size. Additionally, this classification was not validated, which may introduce bias but, importantly, allowed for discovery of new insights that may not have been generated through precoding.

### Conclusions

Routine use of EPPs for online scheduling, patient communication, and telehealth continues to be a critical aspect of care. It is necessary to understand existing disparities in barriers to EPP access to not only improve access to care for all patients but also to continue building patients’ toolbox and self-efficacy to take on active roles in their care. Future research should establish whether interventions, education, and improved eHealth literacy may overcome these barriers.

## Supplementary material

10.2196/72035 Multimedia Appendix 1 Summary of survey questions regarding patient portal.

10.2196/72035 Multimedia Appendix 2 Summary of eHealth Literacy Scale items.

## Abbreviations

ADI: Area Deprivation Index
AOR: adjusted odds ratio
eHEALS: eHealth Literacy Scale
EPP: electronic patient portal
HINTS: Health Information National Trends Survey
HIPAA: Health Insurance Portability and Accountability Act
REDCap: Research Electronic Data Capture
STROBE: Strengthening the Reporting of Observational Studies in Epidemiology
