# Supplementary material for: Assessing the Effects of eHealth Literacy and the Area Deprivation Index on Barriers to Electronic Patient Portal Use for Orthopedic Surgery: Cross-Sectional Observational Study
Source: JMIR Perioper Med. 2026 Jan 7;9:e72035. doi: 10.2196/72035 (PMC12779103; doi:10.2196/72035)
Supplement: Multimedia Appendix 1 [file periop-v9-e72035-s001.docx]

**Multimedia Appendix 1**

Summary of survey questions regarding patient portal.

| 1. **Which of the following are barriers to accessing your patient portal, please ‘check’ those that apply to you (select all that apply).**   *Patients instructed to leave this question blank or to write N/A if no barriers | |
| --- | --- |
|  I do not have internet access | (A1) |
|  I do not need a patient portal | (A2) |
|  I have multiple patient portals | (A3) |
|  Uncomfortable or lack of experience using a computer | (A4) |
|  Prefer to speak directly with a provider/team member by telephone | (A5) |
|  Prefer to speak directly with a provider/team member in-person | (A6) |
|  I have privacy concerns | (A7) |
|  Difficulty logging on/password issues | (A8) |
| 1. **How often (on average) do you access your patient portal?** | |
|  Daily   A few times a week   Once per week   A few times a month   Once per month   A few times a year   Once per year   Never | |

**Inductive coding of barrier type**

Physical access (A1)

Discomfort with technology (A3, A4, A8)

Patient preferences for nonelectronic health care provider communication (A2, A5, A6)
