# Supplementary material for: Assessing the Effects of eHealth Literacy and the Area Deprivation Index on Barriers to Electronic Patient Portal Use for Orthopedic Surgery: Cross-Sectional Observational Study
Source: JMIR Perioper Med. 2026 Jan 7;9:e72035. doi: 10.2196/72035 (PMC12779103; doi:10.2196/72035)
Supplement: Multimedia Appendix 2 [file periop-v9-e72035-s002.docx]

**Multimedia Appendix 2**

Summary of eHEALS scale items.

| Item Number | Strongly Agree | Agree | Undecided | Disagree | Strongly Disagree |
| --- | --- | --- | --- | --- | --- |
| 1. I know what health resources are available on the Internet. | 5 | 4 | 3 | 2 | 1 |
| 2. I know where to find helpful health resources on the Internet. | 5 | 4 | 3 | 2 | 1 |
| 3. I know how to find helpful health resources on the Internet. | 5 | 4 | 3 | 2 | 1 |
| 4. I know how to use the Internet to answer my questions about health. | 5 | 4 | 3 | 2 | 1 |
| 5. I know how to use the health information I find on the Internet to help me. | 5 | 4 | 3 | 2 | 1 |
| 6. I have the skills I need to evaluate the health resources I find on the Internet. | 5 | 4 | 3 | 2 | 1 |
| 7. I can tell high-quality health resources form low-quality health resources on the Internet. | 5 | 4 | 3 | 2 | 1 |
| 8. I feel confident in using information from the Internet to make health decisions. | 5 | 4 | 3 | 2 | 1 |
